# Finite-Time *H*_∞_ Control for Time-Delay Markovian Jump Systems with Partially Unknown Transition Rate via General Controllers

**DOI:** 10.3390/e25030402

**Published:** 2023-02-22

**Authors:** Xikui Liu, Xinye Guo, Wencheng Liu, Yan Li

**Affiliations:** 1 College of Mathematics and Systems Science, Shandong University of Science and Technology, Qingdao 266590, China; 2 Department of Electrical Engineering and Information Technology, Shandong University of Science and Technology, Jinan 250031, China; 3 Department of Fundamental Courses, Shandong University of Science and Technology, Jinan 250031, China

**Keywords:** finite-time boundedness, *H*_∞_ control, Markovian jump system, time-delay

## Abstract

This paper deals with the problems of finite-time boundedness (FTB) and $H_{\infty }$ FTB for time-delay Markovian jump systems with a partially unknown transition rate. First of all, sufficient conditions are provided, ensuring the FTB and $H_{\infty }$ FTB of systems given by linear matrix inequalities (LMIs). A new type of partially delay-dependent controller (PDDC) is designed so that the resulting closed-loop systems are finite-time bounded and satisfy a given $H_{\infty }$ disturbance attenuation level. The PDDC contains both non-time-delay and time-delay states, though not happening at the same time, which is related to the probability distribution of the Bernoulli variable. Furthermore, the PDDC is extended to two other cases; one does not contain the Bernoulli variable, and the other experiences a disordering phenomenon. Finally, three numerical examples are used to show the effectiveness of the proposed approaches.

## 1. Introduction

In actual industrial processes, the transient performance of systems is sometimes particularly important. For example, aircraft control systems require that the states not exceed a given limit [1]; the temperature of a chemical reaction needs to be strictly controlled within a certain range [2]; the angular location of a robot arm should be limited to a particular scope [3]. In recent years, an increasing number of academics have focused on the finite-time stability (FTS) problem. Different from the traditional Lyapunov stability [4,5,6,7], FTS discusses the transient performance of systems in the finite-time interval. In fact, the stable systems in the Lyapunov sense may have very bad transient performances, such as severe oscillation. The definition of FTS (or short-time stability [8]) was first proposed by Kamenkov in [9]. According to FTS, a system state is limited to a certain critical value within a certain time region, if the initial state is norm bounded. The authors of [10] extended FTS to the concept of FTB and took external disturbances into account. The studies of FTS and FTB have been further developed with the evolution of LMI theory [11,12,13,14,15,16,17,18,19]. For example, in [11], sufficient conditions for FTB of closed-loop systems were given in the form of LMIs by designing a dynamic feedback controller. Meanwhile, finite-time $H_{\infty }$ control/filtering problems [20,21,22,23,24] have received much attention in order to reduce influences on a system caused by external disturbances.

On the other hand, abrupt changes are often encountered in the industrial process due to a component fault, invalidation, an associated change between subsystems, a sudden environmental disturbance [25], and so on. The occurrence of these situations causes the structure and parameters of a system to switch between various subsystems, such as networked control systems or power electrics. The Markov jump system [26] is used to deal with this kind of practical system via the transition probabilities of the jump process. In recent decades, many researchers have performed studies on these types of systems, such as [27,28,29,30,31,32]. Moreover, some research results have been utilized in many engineering fields, such as power systems [33], manufacturing systems [34], communication systems [35], etc. Although there have been lots of research achievements about Markov jump systems, most assume that the transition probabilities are all known; however, it is difficult to ascertain precise transition probabilities in real life due to instrument and measurement limitations. Therefore, further research on a Markov jump system with a partially unknown transition rate is really vital and necessary. Readers may refer to [36,37,38].

Time delay, as an inevitable phenomenon, widely exists in communication [39], the chemical industry [40], transportation [41], and other systems. The existence of a time delay may make the performance of a system deteriorate, destroy the balance and stability of systems, and even produce a chaos phenomenon. This leads to the development of and changes to the systems such that they depend not only on the present state but also on the previous state [42,43,44,45,46]. By studying the FTS of various time-delay systems [47,48,49,50], it is found that most of the results mainly consider the controller with or without a time delay. However, in practice, data transmission events with or without a delay occur randomly, which inspires us to consider designing a controller with both a time delay and no time delay, or non-simultaneous occurrences according to probability.

In this paper, we handle the FTB and $H_{\infty }$ FTB problems of time-delay Markov jump systems with a partially unknown transition rate via some general PDDCs. The following are the main contributions: (1) With LMIs, we give sufficient conditions for FTB for the defined system. (2) A new kind of PDDC is designed to make the resulting system $H_{\infty }$ FTB. The PDDC contains both non-time-delay and time-delay states; however, these are not happening at the same time. In comparison to conventional state feedback controllers [47,48,49,51], the probability distributions play an important role in the PDDC. (3) Different from the existing results of [46], the PDDC is extended to two new cases: one does not contain the Bernoulli variable, and the other experiences a disordering phenomenon.

The rest of this paper is arranged as follows: In Section 2, the preparation and problem statement are presented. Section 3 discusses the main results for the FTB and $H_{\infty }$ FTB of the system defined by LMIs via the PDDC’s design. Three examples are given to show the effectiveness of the obtained results in Section 4. Some conclusions are given in Section 5.

Notation: $\lambda_{\max}\left(Q\right)$ ($\lambda_{\min}\left(Q\right)$) means the maximum (minimum) eigenvalue of a real symmetric matrix *Q*; E[·] refers to the mathematical expectation operator; the superscript *T* is the transposition of the matrix. In the matrices, diag {⋯} stands for the block-diagonal matrix, the symbol ∗ is the symmetric term of a matrix, and ${\left(P\right)}^{🟉}=P+P^{T}$. The σ-algebras of the sample space subsets are represented by F. Pr denotes the mathematic probability.

## 2. Problem Statement and Preliminaries

Consider a linear time-delay Itô stochastic Markovian switching system

(1) $$\left\{ \begin{array}{cc} dx(t)= & \;[S\left(\sigma_{t}\right)x\left(t\right)+S_{\tau }\left(\sigma_{t}\right)x\left(t-\tau \right)+L\left(\sigma_{t}\right)u\left(t\right)+G\left(\sigma_{t}\right)v\left(t\right)]dt\\ & +\left[U\left(\sigma_{t}\right)x\left(t\right)+U_{\tau }\left(\sigma_{t}\right)x\left(t-\tau \right)+J\left(\sigma_{t}\right)u\left(t\right)+F\left(\sigma_{t}\right)v\left(t\right)\right]d\omega \left(t\right),\\ z\left(t\right)= & H\left(\sigma_{t}\right)x\left(t\right)+H_{\tau }\left(\sigma_{t}\right)x\left(t-\tau \right)+D\left(\sigma_{t}\right)v\left(t\right),\;\forall t\in \left[0,\tilde{T}\right],\\ x\left(t\right)= & \psi (t),\;\sigma_{t}=\sigma_{0},\;\forall t\in [-\tau ,\;0], \end{array}\right.$$

where $x\left(t\right)\in R^{n}$ is the system state, $u\left(t\right)\in R^{m}$ is the control input, and $z\left(t\right)\in R^{q}$ is the control output. $S(\sigma_{t})$, $S_{\tau }\left(\sigma_{t}\right)$, $L(\sigma_{t})$, $G(\sigma_{t})$, $U(\sigma_{t})$, $U_{\tau }\left(\sigma_{t}\right)$, $J(\sigma_{t})$, $F(\sigma_{t})$, $H(\sigma_{t})$, $H_{\tau }\left(\sigma_{t}\right)$, and $D(\sigma_{t})$ are constant matrices, for simplicity. When $\sigma_{t}=i$, they are denoted as $S_{i}$, $S_{\tau i}$, $L_{i}$, $G_{i}$, $U_{i}$, $U_{\tau i}$, $J_{i}$, $F_{i}$, $H_{i}$, $H_{\tau i}$, and $D_{i}$. The time delay is τ≥0. The continuous vector-valued function ψ(t) is defined on [−τ,0]; ω(t) is the standard one-dimensional Wiener process defined on the probability space (Ω,F,P) satisfying $E\left[d\omega \left(t\right)\right]=0,E\left[d^{2}\omega \left(t\right)\right]=dt$; and v(t) is the external disturbance satisfying

(2) $$\begin{array}{c} \int_{0}^{t}v^{T}\left(s\right)v\left(s\right)ds<d^{2},\;d>0. \end{array}$$

The transition rate of the Markovian process $\left\{\sigma_{t},t\geq 0\right\}$ is given by

(3) $$\begin{array}{c} Pr\left(\sigma_{t+▵t}=j|\sigma_{t}=i\right)=\left\{\begin{array}{c} \pi_{ij}▵t+o\left(▵t\right),i\neq j,\\ 1+\pi_{ii}▵t+o\left(▵t\right),i=j, \end{array}\right. \end{array}$$

where $\left\{\sigma_{t},t\geq 0\right\}$ takes the values in S={1,2,⋯,N}, o(▵t) is the order of ▵t that satisfies $▵t>0,and\lim_{▵t\to 0}\frac{o(▵t)}{▵t}=0$. $\pi_{ij}\geq 0\left(i\neq j,i,j\in S\right)$ is the transition rate of σ(t) from the mode *i* at the time *t* to the mode *j* at the time t+▵t, such that $\pi_{ii}=-\sum_{j\neq i}\pi_{ij}$. All of the transition rates $\pi_{ij},i,j\in S$, can be collected into the following transition rate matrix

$$\Pi =\left[\begin{array}{cccc} \pi_{11} & \pi_{12} & \cdots & \pi_{1N}\\ \pi_{21} & \pi_{22} & \cdots & \pi_{2N}\\ \vdots & \cdots & \ddots & \vdots \\ \pi_{N1} & \pi_{N2} & \cdots & \pi_{NN} \end{array}\right].$$

Assume that the transition rate is partially unknown, for example, there is a 2 × 2 transition rate matrix

$$\begin{array}{c} \Pi_{1}=\left[\begin{array}{cc} \pi_{11} & \pi_{12}\\ ? & ? \end{array}\right] \end{array}$$

where “?” is an unknown element and $\pi_{ij}$ is known. For all $\pi_{ij}\in S$, define $S=L_{k}^{i}+L_{uk}^{i}$, where

$$\begin{array}{c} L_{k}^{i}=\left\{j:\;\mathrm{if}\;\pi_{ij}\;\mathrm{is}\;\mathrm{known}\right\},\;L_{uk}^{i}=\left\{j:\;\mathrm{if}\;\pi_{ij}\;\mathrm{is}\;\mathrm{unknown}\right\}. \end{array}$$

If $L_{k}^{i}$ is non-empty, it is described as follows

$$\begin{array}{c} L_{k}^{i}=\left\{k_{1}^{i},\;k_{2}^{i},\;\cdots ,\;k_{m}^{i},\right\},\;0\leq m\leq N, \end{array}$$

where $k_{m}^{i}\in S$ denotes the *m*th known element in the matrix Π’s *i*th row.

**Definition 1** (FTB)**.** *For the given scalars $c_{2}>c_{1}>0$, $\tilde{T}>0$ and the matrix $R_{i}>0\;\left(i\in S\right)$, system (1) with u(t)=0 is FTB with respect to $\left(c_{1},c_{2},\tilde{T},R_{i},d\right)$, if*

(4) $$\begin{array}{c} \begin{array}{c} E\left[x^{T}\left(t_{1}\right)R_{i}x\left(t_{1}\right)\right]\leq c_{1}\Rightarrow E\left[x^{T}\left(t_{2}\right)R_{i}x\left(t_{2}\right)\right]<c_{2}, \end{array} \end{array}$$

*and (2) holds, where $t_{1}\in \left[-\tau ,0\right],\;t_{2}\in \left[0,\tilde{T}\right]$.*

**Remark** **1.** *FTB can be simplified to FTS with respect to $\left(c_{1},c_{2},\tilde{T},R_{i}\right)$ when v(t)=0. The FTB/FTS can be used to solve some practical problems, such as the chemical reaction process, electronic circuit systems, and medicine. For example, the body’s normal systolic blood pressure is 90–140 mmHg. If the body’s systolic blood pressure is greater than 140 mmHg, then one suffers from high blood pressure disease. One must take blood pressure medicine.*

**Definition 2** (*H*_∞_ FTB)**.** *For the given scalar γ>0, system (1) with u(t)=0 is $H_{\infty }$ FTB with respect to $\left(c_{1},c_{2},\tilde{T},R_{i},d,\gamma \right)$. If system (1) is FTB and under zero initial condition, for any non-zero disturbance v(t), the control output z(t) satisfies*

(5) $$\begin{array}{c} \begin{array}{c} E\left[\int_{0}^{\tilde{T}}z^{T}\left(t\right)z\left(t\right)dt\right]<\gamma^{2}E\left[\int_{0}^{\tilde{T}}v^{T}\left(t\right)v\left(t\right)dt\right]. \end{array} \end{array}$$

When the control problem is considered, the following definition is needed.

**Definition 3** (*H*_∞_ FTB stabilization)**.** *System (1) is finite-time $H_{\infty }$ stabilizable if there exists a controller u(t) such that the resulting closed-loop system is $H_{\infty }$ FTB.*

**Lemma** **1** (Gronwall–Bellman inequality [52,53]). *Let g(t) be a nonnegative continuous function. If there are positive constants r,q such that*

(6) $$\begin{array}{c} \begin{array}{c} g\left(t\right)\leq r+q\int_{0}^{t}g\left(s\right)ds,\;0\leq t\leq \tilde{T}, \end{array} \end{array}$$

*then*

(7) $$\begin{array}{c} \begin{array}{c} g\left(t\right)\leq r\exp(qt),\;0\leq t\leq \tilde{T}. \end{array} \end{array}$$

**Remark** **2.** *Lemma 1 can be reformulated with sharp inequalities. The proof is given in Appendix A*.

**Lemma** **2** (Schur’s complement lemma [54]). *For the real matrix H, the real symmetric matrix S, and the positive-definite matrix U, the below inequalities are equivalent:*

$$\begin{array}{c} \begin{array}{c} S+HU^{-1}H^{T}<0 \end{array} \end{array}$$

*and*

$$\begin{array}{c} \left[\begin{array}{cc} S & H\\ H^{T} & -U \end{array}\right]<0. \end{array}$$

## 3. Main Results

Firstly, we discuss the FTB problem for system (1) (when u(t)=0) in this section.

**Theorem** **1.** *System (1) (when u(t)=0) is FTB with respect to $\left(c_{1},c_{2},\tilde{T},R_{i},d\right)$, if for a real scalar η≥0, there exist the scalars $\lambda_{i1}>0$, $\lambda_{i2}>0$, symmetric matrices $P_{i}>0,$ $Q_{i}>0$, and $O_{i}>0$ satisfying*

(8) $$\begin{array}{c} \left[\begin{array}{cccc} \Psi_{i1} & P_{i}S_{\tau i} & P_{i}G_{i} & U_{i}^{T}\\ {}* & -O_{i} & 0 & U_{\tau i}^{T}\\ {}* & * & -Q_{i} & F_{i}^{T}\\ {}* & * & * & -P_{i}^{-1} \end{array}\right]<0, \end{array}$$

(9) $$\begin{array}{c} O_{i}<R_{i}, \end{array}$$

(10) $$\begin{array}{c} \lambda_{i1}I<{\bar{P}}_{i}<\lambda_{i2}I, \end{array}$$

(11) $$\begin{array}{c} c_{1}\left(\lambda_{i2}+\tau \right)+\lambda_{\max}\left(Q_{i}\right)d^{2}<c_{2}exp\left(-\eta \tilde{T}\right)\lambda_{i1}, \end{array}$$

*where*

$$\begin{array}{cc} \Psi_{i1}= & \sum_{j\in L_{uk}^{i}}\pi_{ij}\left[{\left(P_{i}S_{i}\right)}^{🟉}+O_{i}+P_{j}\right]+\zeta_{k}^{i}+\left(1+\pi_{k}^{i}\right)\left[{\left(P_{i}S_{i}\right)}^{🟉}+O_{i}\right],\\ \zeta_{k}^{i}= & \sum_{j\in L_{k}^{i}}\pi_{ij}P_{j},\;\pi_{k}^{i}=\sum_{j\in L_{k}^{i}}\pi_{ij},\;{\bar{P}}_{i}=R_{i}^{-\frac{1}{2}}P_{i}R_{i}^{-\frac{1}{2}}. \end{array}$$

**Proof.**  For system (1), we choose a stochastic Lyapunov functional as

(12) $$\begin{array}{c} \begin{array}{c} V\left(x_{t},\sigma_{t}\right)=x^{T}\left(t\right)P\left(\sigma_{t}\right)x\left(t\right)+\int_{t-\tau }^{t}x^{T}\left(s\right)O\left(\sigma_{t}\right)\left(s\right)x\left(s\right)ds. \end{array} \end{array}$$

For each $\sigma_{t}=i\in S,$ let L be the differential generating operator of system (1). According to the Itô formula, it follows that

(13) $$\begin{array}{c} \begin{array}{cc} & LV(x_{t},\sigma_{t}=i)\\ = & \;x^{T}\left(t\right)[{\left(P_{i}S_{i}\right)}^{🟉}+\sum_{j=1}^{N}\pi_{ij}P_{j}+O_{i}]x\left(t\right)+{\left[\Xi \right]}^{T}P_{i}\left[\Xi \right]-x^{T}\left(t-\tau \right)O_{i}x\left(t-\tau \right)\\ & +2x^{T}\left(t\right)P_{i}S_{\tau i}x\left(t-\tau \right)+2x^{T}\left(t\right)P_{i}G_{i}v\left(t\right)\\ = & \;x^{T}\left(t\right)\left[{\left(P_{i}S_{i}\right)}^{🟉}+O_{i}+\sum_{j\in L_{uk}^{i}}\pi_{ij}P_{j}+\zeta_{k}^{i}+\sum_{j\in L_{k}^{i}}\pi_{ij}\left({\left(P_{i}S_{i}\right)}^{🟉}+O_{i}\right)\right]x\left(t\right)\\ & +2x^{T}\left(t\right)P_{i}\left[S_{\tau i}x\left(t-\tau \right)+G_{i}v\left(t\right)\right]+{\left[\Xi \right]}^{T}P_{i}\left[\Xi \right]-x^{T}\left(t-\tau \right)O_{i}x\left(t-\tau \right)\\ = & \;x^{T}\left(t\right)\left[\left(1+\pi_{k}^{i}\right)\left({\left(P_{i}S_{i}\right)}^{🟉}+O_{i}\right)+\zeta_{k}^{i}+\sum_{j\in L_{uk}^{i}}\pi_{ij}\left({\left(P_{i}S_{i}\right)}^{🟉}+O_{i}+P_{j}\right)\right]x\left(t\right)\\ & +2x^{T}\left(t\right)P_{i}\left[S_{\tau i}x\left(t-\tau \right)+G_{i}v\left(t\right)\right]+{\left[\Xi \right]}^{T}P_{i}\left[\Xi \right]-x^{T}\left(t-\tau \right)O_{i}x\left(t-\tau \right) \end{array} \end{array}$$

where $\Xi =F_{i}v\left(t\right)+U_{i}x\left(t\right)+U_{\tau i}x\left(t-\tau \right)$.

From (8) and (13), it is easy to obtain

$$\begin{array}{c} LV\left(x_{t},\sigma_{t}=i\right)<\eta V_{1}\left(x_{t},\sigma_{t}=i\right)+v^{T}\left(t\right)Q_{i}v\left(t\right),\forall t\in \left[0,\tilde{T}\right], \end{array}$$

where $V_{1}\left(x_{t},\sigma_{t}=i\right)=x^{T}\left(t\right)P_{i}x\left(t\right)$, so

(14) $$\begin{array}{c} LV\left(x_{t},\sigma_{t}=i\right)<\eta V\left(x_{t},\sigma_{t}=i\right)+\lambda_{\max}\left(Q_{i}\right)v^{T}\left(t\right)v\left(t\right). \end{array}$$

Integrating both sides of (14) from 0 to *t*$\left(t\in \left[0,\tilde{T}\right]\right)$ yields

(15) $$\begin{array}{c} \begin{array}{c} V\left(x_{t},\sigma_{t}=i\right)-V\left(x_{0},\sigma_{0}\right)<\eta \int_{0}^{t}V\left(x_{s},\sigma_{s}\right)ds+\lambda_{\max}\left(Q_{i}\right)\int_{0}^{t}v^{T}\left(s\right)v\left(s\right)ds. \end{array} \end{array}$$

Taking the mathematical expectation on both sides of (15), the following is concluded

$$\begin{array}{c} \begin{array}{c} E\left[V\left(x_{t},\sigma_{t}=i\right)\right]-E\left[V\left(x_{0},\sigma_{0}\right)\right]<\eta E\left[\int_{0}^{t}V\left(x_{s},\sigma_{s}\right)ds\right]+\lambda_{\max}\left(Q_{i}\right)E\left[\int_{0}^{t}v^{T}\left(s\right)v\left(s\right)ds\right], \end{array} \end{array}$$

i.e.,

(16) $$\begin{array}{c} \begin{array}{c} E\left[V\left(x_{t},\sigma_{t}=i\right)\right]<E\left[V\left(x_{0},\sigma_{0}\right)\right]+\eta \int_{0}^{t}E\left[V\left(x_{s},\sigma_{s}\right)\right]ds+\lambda_{\max}\left(Q_{i}\right)E\left[\int_{0}^{t}v^{T}\left(s\right)v\left(s\right)\right]ds. \end{array} \end{array}$$

Applying Lemma 1 or the Gronwall–Bellman-type inequality for the three functions [55] to (16) yields

(17) $$\begin{array}{c} \begin{array}{c} E\left[V\left(x_{t},\sigma_{t}=i\right)\right]<E\left[V\left(x_{0},\sigma_{0}\right)\right]\exp(\eta t)+\lambda_{\max}\left(Q_{i}\right)E\left[\int_{0}^{t}v^{T}\left(s\right)v\left(s\right)ds\right]\exp(\eta t). \end{array} \end{array}$$

Set ${\overset{˘}{\lambda }}_{i}=\min_{i\in S}\lambda_{\min}\left({\bar{P}}_{i}\right)$ and ${\hat{\lambda }}_{i}=\max_{i\in S}\lambda_{\max}\left({\bar{P}}_{i}\right)$. Together with (10), we have

(18) $$\begin{array}{c} \begin{array}{cc} & E\left[V\left(x_{t},\sigma_{t}=i\right)\right]=\;E\left[\int_{t-\tau }^{t}x^{T}\left(s\right)O_{i}x\left(s\right)ds\right]+E\left[V_{1}\left(x_{t},\sigma_{t}=i\right)\right]\\ \geq & \;E\left[V_{1}\left(x_{t},\sigma_{t}=i\right)\right]\geq {\overset{˘}{\lambda }}_{i}E\left[x^{T}\left(t\right)R_{i}x\left(t\right)\right]\geq \lambda_{i1}E\left[x^{T}\left(t\right)R_{i}x\left(t\right)\right], \end{array} \end{array}$$

(19) $$\begin{array}{c} \begin{array}{cc} & E\left[V\left(x_{0},\sigma_{0}=i\right)\right]\exp\left(\eta t\right)=E\left[x^{T}\left(0\right)R_{i}^{\frac{1}{2}}{\bar{P}}_{i}R_{i}^{\frac{1}{2}}x\left(0\right)\right]\exp\left(\eta t\right)+E\left[\int_{-\tau }^{0}x^{T}\left(s\right)O_{i}x\left(s\right)ds\right]\exp\left(\eta t\right)\\ \leq & \;c_{1}\left({\hat{\lambda }}_{i}+\tau \right)\exp\left(\eta \tilde{T}\right)\leq c_{1}\left(\lambda_{i2}+\tau \right)\exp\left(\eta \tilde{T}\right), \end{array} \end{array}$$

(20) $$\begin{array}{c} \lambda_{\max}\left(Q_{i}\right)E\left[\int_{0}^{t}v^{T}\left(s\right)v\left(s\right)ds\right]\exp\left(\eta t\right)<\lambda_{\max}\left(Q_{i}\right)d^{2}\exp\left(\eta \tilde{T}\right). \end{array}$$

From conditions (17) to (20), it is derived

(21) $$E\left[x^{T}\left(t\right)R_{i}x\left(t\right)\right]\leq \exp\left(\eta \tilde{T}\right)\left[\frac{\left(\tau +\lambda_{i2}\right)c_{1}+\lambda_{\max}\left(Q_{i}\right)d^{2}}{\lambda_{i1}}\right].$$

For all $t\in \left[0,\tilde{T}\right],E\left[x^{T}\left(t\right)R_{i}x\left(t\right)\right]<c_{2}$ holds, which is obtained by

$$\left[c_{1}\left(\lambda_{i2}+\tau \right)+\lambda_{\max}\left(Q_{i}\right)d^{2}\right]\exp\left(\eta \tilde{T}\right)\lambda_{i1}^{-1}<c_{2},$$

which is (11). The proof is complete. □

**Remark** **3.** *If $F_{i}=G_{i}=0$, then Theorem 1 is reduced to Theorem 1 in [29].*

In the following, we propose three novel types of partially delay-dependent controllers. One of the controllers is

(22) $$\begin{array}{c} u\left(t\right)=\left(1-\delta \left(t\right)\right)K_{\tau }\left(\sigma_{t}\right)x\left(t-\tau \right)+\delta \left(t\right)K\left(\sigma_{t}\right)x\left(t\right), \end{array}$$

where $K_{\tau }\left(\sigma_{t}\right)$ and $K(\sigma_{t})$ represent the control gains, and δ(t) is the Bernoulli variable defined as

$$\begin{array}{c} \delta \left(t\right)=\left\{\begin{array}{c} 1,\;\mathrm{if}\;x(t)\;\mathrm{is}\;\mathrm{available},\\ 0,\;\mathrm{if}\;x(t-\tau )\;\mathrm{is}\;\mathrm{available}, \end{array}\right. \end{array}$$

and satisfies

$$\begin{array}{c} Pr\{\delta (t)=1\}=\delta ,\;Pr\{\delta (t)=0\}=1-\delta . \end{array}$$

Furthermore,

$$\begin{array}{c} E\left[{\left(\delta \left(t\right)-\delta \right)}^{2}\right]=\delta \left(1-\delta \right)=\beta^{2},\;E\left[\delta \left(t\right)-\delta \right]=0. \end{array}$$

Substituting (22) in (1), we have

(23) $$\begin{array}{c} \left\{\begin{array}{cc} dx(t)= & [\hat{S}\left(\sigma_{t}\right)x\left(t\right)+{\hat{S}}_{\tau }\left(\sigma_{t}\right)x\left(t-\tau \right)+G\left(\sigma_{t}\right)v\left(t\right)+\left(\delta \left(t\right)-\delta \right)W\left(\sigma_{t}\right)]dt\\ & +\left[\hat{U}\left(\sigma_{t}\right)x\left(t\right)+{\hat{U}}_{\tau }\left(\sigma_{t}\right)x\left(t-\tau \right)+F\left(\sigma_{t}\right)v\left(t\right)+\left(\delta \left(t\right)-\delta \right)Z\left(\sigma_{t}\right)\right]d\omega \left(t\right),\\ z(t)= & \;H\left(\sigma_{t}\right)x\left(t\right)+H_{\tau }\left(\sigma_{t}\right)x\left(t-\tau \right)+D\left(\sigma_{t}\right)v\left(t\right),\;\forall t\in \left[0,\tilde{T}\right],\\ x(t)= & \;\psi \left(t\right),\;\sigma_{t}=\sigma_{0},\;\forall t\in \left[-\tau ,0\right], \end{array}\right. \end{array}$$

where

$$\begin{array}{cc} \hat{S}\left(\sigma_{t}\right)= & \;S\left(\sigma_{t}\right)+\delta L\left(\sigma_{t}\right)K\left(\sigma_{t}\right),\;{\hat{S}}_{\tau }\left(\sigma_{t}\right)=S_{\tau }\left(\sigma_{t}\right)+\left(1-\delta \right)L\left(\sigma_{t}\right)K_{\tau }\left(\sigma_{t}\right),\\ W(\sigma_{t})= & \;-L\left(\sigma_{t}\right)K_{\tau }\left(\sigma_{t}\right)x\left(t-\tau \right)+T\left(\sigma_{t}\right)K\left(\sigma_{t}\right)x\left(t\right),\;\hat{U}\left(\sigma_{t}\right)=U\left(\sigma_{t}\right)+\delta J\left(\sigma_{t}\right)K\left(\sigma_{t}\right),\\ {\hat{U}}_{\tau }\left(\sigma_{t}\right)= & \;U_{\tau }\left(\sigma_{t}\right)+\left(1-\delta \right)J\left(\sigma_{t}\right)K_{\tau }\left(\sigma_{t}\right),\;Z\left(\sigma_{t}\right)=-J\left(\sigma_{t}\right)K_{\tau }\left(\sigma_{t}\right)x\left(t-\tau \right)+J\left(\sigma_{t}\right)K\left(\sigma_{t}\right)x\left(t\right). \end{array}$$

The following theorem gives the sufficient condition of $H_{\infty }$ FTB for the closed-loop system (23) via controller (22).

**Theorem** **2.** *System (23) is $H_{\infty }$ FTB with respect to $\left(c_{1},c_{2},\tilde{T},R_{i},d,\gamma \right)$, if for a real scalar η≥0, there exist the scalars γ>0, $\lambda_{i1}>0$, $\lambda_{i2}>0$, matrices $X_{i}>0$, ${\bar{O}}_{i}>0$, and $Y_{i}$, $Y_{\tau i}$ satisfying*

(24) $$\left[\begin{array}{ccccccc} {\tilde{\Psi }}_{i1} & {\tilde{\Psi }}_{i2} & G_{i}X_{i} & H_{i}^{T} & {\tilde{\Psi }}_{i3} & {\tilde{\Psi }}_{i4} & X_{i}\\ {}* & {\tilde{\Psi }}_{i5} & 0 & H_{\tau i}^{T} & {\tilde{\Psi }}_{i6} & {\tilde{\Psi }}_{i7} & 0\\ {}* & * & -\gamma^{2}I & D_{i} & F_{i}^{T} & 0 & 0\\ {}* & * & * & -I & 0 & 0 & 0\\ {}* & * & * & * & -X_{i} & 0 & 0\\ {}* & * & * & * & * & -X_{i} & 0\\ {}* & * & * & * & * & * & -{\bar{O}}_{i} \end{array}\right]<0,$$

(25) $$\begin{array}{c} R_{i}^{-1}<{\bar{O}}_{i}, \end{array}$$

(26) $$\begin{array}{c} \left[\begin{array}{cc} -\lambda_{i1} & R_{i}^{-\frac{1}{2}}\\ \;* & -X_{i} \end{array}\right]<0, \end{array}$$

(27) $$\begin{array}{c} -2R_{i}^{-\frac{1}{2}}+X_{i}+\lambda_{i1}I<0, \end{array}$$

(28) $$\begin{array}{c} c_{1}\left(\lambda_{i2}+\tau \right)+\gamma^{2}d^{2}<c_{2}e^{-\eta \tilde{T}}\lambda_{i1}, \end{array}$$

*where*

$$\begin{array}{cc} {\tilde{\Psi }}_{i1}= & \;\left(1+X_{i}\pi_{k}^{i}\right){\left(S_{i}X_{i}+\delta L_{i}Y_{i}\right)}^{🟉}+X_{i}\zeta_{k}^{i}X_{i}^{T}\\ \; & \;+X_{i}\sum_{j\in L_{uk}^{i}}\pi_{ij}\left[{\left(S_{i}X_{i}+\delta L_{i}Y_{i}\right)}^{🟉}+X_{j}^{-1}X_{i}^{T}\right]-\eta X_{i},\\ {\tilde{\Psi }}_{i2}= & \;S_{\tau i}X_{i}+\left(1-\delta \right)L_{i}Y_{\tau i},\;{\tilde{\Psi }}_{i3}=X_{i}U_{i}^{T}+\delta Y_{i}^{T}J_{i}^{T},\;{\tilde{\Psi }}_{i4}=\beta Y_{i}^{T}J_{i}^{T},\\ {\tilde{\Psi }}_{i5}= & -2X_{i}+{\bar{O}}_{i},\;{\tilde{\Psi }}_{i6}=X_{i}U_{\tau i}^{T}+\left(1-\delta \right)Y_{\tau i}^{T}J_{i}^{T},\;{\tilde{\Psi }}_{i7}=-\beta Y_{\tau i}^{T}J_{i}^{T}. \end{array}$$

*Moreover, the gains of controller (22) are*

$$K_{i}=Y_{i}X_{i}^{-1},\;K_{\tau i}=Y_{\tau i}X_{i}^{-1}.$$

**Proof.**  Choosing the Lyapunov functional (12) for system (23), we obtain

(29) $$\begin{array}{c} \begin{array}{cc} & LV(x_{t},\sigma_{t}=i)\\ = & \;x^{T}\left(t\right)\left[{\left(P_{i}{\hat{S}}_{i}\right)}^{🟉}+\sum_{j=1}^{N}\pi_{ij}P_{j}\right]x\left(t\right)+2x^{T}\left(t\right)P_{i}G_{i}v\left(t\right)+2x^{T}\left(t\right)P_{i}{\hat{S}}_{\tau i}x\left(t-\tau \right)\\ & +x^{T}\left(t\right)O_{i}x\left(t\right)-x^{T}\left(t-\tau \right)O_{i}x\left(t-\tau \right)+\beta^{2}Z_{i}^{T}P_{i}Z_{i}+{\tilde{\Xi }}^{T}P_{i}\tilde{\Xi }\\ = & \;x^{T}\left(t\right)\left[\left(1+\pi_{k}^{i}\right){\left(P_{i}{\hat{S}}_{i}\right)}^{🟉}+\zeta_{k}^{i}+\sum_{j\in L_{uk}^{i}}\pi_{ij}\left({\left(P_{i}{\hat{S}}_{i}\right)}^{🟉}+P_{j}\right)+O_{i}\right]x\left(t\right)\\ & +2x^{T}\left(t\right)P_{i}G_{i}v\left(t\right)+\beta^{2}Z_{i}^{T}P_{i}Z_{i}+{\tilde{\Xi }}^{T}P_{i}\tilde{\Xi }\\ & -x^{T}\left(t-\tau \right)O_{i}x\left(t-\tau \right)+2x^{T}\left(t\right)P_{i}{\hat{S}}_{\tau i}x\left(t-\tau \right), \end{array} \end{array}$$

where $\tilde{\Xi }=F_{i}v\left(t\right)+{\hat{U}}_{i}x\left(t\right)+{\hat{U}}_{\tau i}x\left(t-\tau \right)$.

Let ${\bar{O}}_{i}=O_{i}^{-1}$ per (24) and the following inequality

(30) $$\begin{array}{c} -X_{i}O_{i}X_{i}\leq -X_{i}-X_{i}+{\bar{O}}_{i},. \end{array}$$

The following result is obtained

(31) $$\left[\begin{array}{ccccccc} {\tilde{\Psi }}_{i1} & {\tilde{\Psi }}_{i2} & G_{i}X_{i} & H_{i}^{T} & {\tilde{\Psi }}_{i3} & {\tilde{\Psi }}_{i4} & X_{i}\\ {}* & {\overset{´}{\Psi }}_{i5} & 0 & H_{\tau i}^{T} & {\tilde{\Psi }}_{i6} & {\tilde{\Psi }}_{i7} & 0\\ {}* & * & -\gamma^{2}I & D_{i} & F_{i}^{T} & 0 & 0\\ {}* & * & * & -I & 0 & 0 & 0\\ {}* & * & * & * & -X_{i} & 0 & 0\\ {}* & * & * & * & * & -X_{i} & 0\\ {}* & * & * & * & * & * & -{\bar{O}}_{i} \end{array}\right]<0,$$

where ${\overset{´}{\Psi }}_{i5}=-X_{i}O_{i}X_{i}$.

By pre- and post-multiplying both sides of (31), respectively, by diag $\left\{X_{i}^{-1},X_{i}^{-1},I,I,I,I,I\right\}$ and diag ${\left\{X_{i}^{-1},X_{i}^{-1},I,I,I,I,I\right\}}^{T}$; denoting $X_{i}=P_{i}^{-1},Y_{i}=K_{i}X_{i},Y_{\tau i}=K_{\tau i}X_{i}$; and according to Lemma 2, one obtains

(32) $$\begin{array}{c} \left[\begin{array}{ccc} \Pi_{i1} & \Pi_{i2} & \Pi_{i3}\\ {}* & -\gamma^{2}I+F_{i}^{T}P_{i}F_{i} & D_{i}\\ {}* & * & -I \end{array}\right]<0,\; \end{array}$$

where

$$\begin{array}{cc} \Pi_{i1}= & \left[\begin{array}{cc} {\hat{\Omega }}_{i1} & {\hat{\Omega }}_{i2}\\ {}* & {\hat{\Omega }}_{i3} \end{array}\right],\\ {\hat{\Omega }}_{i1}= & \;\left(1+\pi_{k}^{i}\right){\left(P_{i}S_{i}+\delta P_{i}L_{i}K_{i}\right)}^{🟉}+\zeta_{k}^{i}\\ \; & +\sum_{j\in L_{uk}^{i}}\pi_{ij}\left({\left(P_{i}S_{i}+\delta P_{i}L_{i}K_{i}\right)}^{🟉}+P_{j}\right)+O_{i}-\eta P_{i}\\ \; & +{\left(U_{i}+\delta J_{i}K_{i}\right)}^{T}P_{i}\left(U_{i}+\delta J_{i}K_{i}\right)+\beta^{2}{\left(J_{i}K_{i}\right)}^{T}P_{i}\left(J_{i}K_{i}\right),\\ {\hat{\Omega }}_{i2}= & \;P_{i}S_{\tau i}+P_{i}\left(L_{i}K_{\tau }i\right)+\beta^{2}{\left(J_{i}K_{i}\right)}^{T}P_{i}\left(J_{i}K_{\tau i}\right)\\ \; & +{\left(U_{i}+\delta J_{i}K_{i}\right)}^{T}P_{i}\left(U_{\tau i}+\left(1-\delta \right)J_{i}K_{\tau i}\right),\\ {\hat{\Omega }}_{i3}= & -O_{i}+\beta^{2}{\left(J_{i}K_{\tau i}\right)}^{T}P_{i}\left(J_{i}K_{\tau i}\right)\\ \; & +{\left(U_{\tau i}+\delta J_{i}K_{\tau i}\right)}^{T}P_{i}\left(U_{\tau i}+\left(1-\delta \right)J_{i}K_{\tau i}\right),\\ \Pi_{i2}= & \left[\begin{array}{c} P_{i}G_{i}+{\left(U_{i}+\delta J_{i}K_{i}\right)}^{T}P_{i}F_{i}\\ {\left(U_{\tau i}+\left(1-\delta \right)J_{i}K_{\tau i}\right)}^{T}P_{i}F_{i} \end{array}\right],\;\Pi_{i3}={\left[\begin{array}{cc} H_{i} & H_{\tau i} \end{array}\right]}^{T}. \end{array}$$

By pre- and post-multiplying (32) by diag $\left[x^{T}\left(t\right)\;x^{T}\left(t-\tau \right)\;v^{T}\left(t\right)\;z^{T}\left(t\right)\right]$ and its transpose, respectively, and comparing it with (29), it is seen that

$$\begin{array}{c} LV\left(x_{t},\sigma_{t}=i\right)<\eta V_{1}\left(x_{t},\sigma_{t}=i\right)+\gamma^{2}v^{T}\left(t\right)v\left(t\right)-z^{T}\left(t\right)z\left(t\right). \end{array}$$

Then, one has

(33) $$\begin{array}{c} \begin{array}{c} LV\left(x_{t},\sigma_{t}=i\right)<\eta V\left(x_{t},\sigma_{t}=i\right)+\gamma^{2}v^{T}\left(t\right)v\left(t\right)-z^{T}\left(t\right)z\left(t\right). \end{array} \end{array}$$

Under zero initial condition, taking mathematical expectation, and integrating both sides of (33) from 0 to *t*$\left(t\in \left[0,\tilde{T}\right]\right)$, by applying Lemma 1, it is deduced that

(34) $$\begin{array}{c} \begin{array}{c} E\left[V\left(x_{t},\sigma_{t}=i\right)\right]<e^{\eta \tilde{T}}\left\{\gamma^{2}E\left[\int_{0}^{\tilde{T}}v^{T}\left(t\right)v\left(t\right)dt\right]-E\left[\int_{0}^{\tilde{T}}z^{T}\left(t\right)z\left(t\right)dt\right]\right\}. \end{array} \end{array}$$

It is also clear that (34) implies

$$E\left[\int_{0}^{\tilde{T}}z^{T}\left(t\right)z\left(t\right)dt\right]<\gamma^{2}E\left[\int_{0}^{\tilde{T}}v^{T}\left(t\right)v\left(t\right)dt\right].$$

By (33), we obtain

(35) $$LV\left(x_{t},\sigma_{t}=i\right)<\eta V\left(x_{t},\sigma_{t}=i\right)+\gamma^{2}v^{T}\left(t\right)v\left(t\right).$$

Because of $R_{i}>0$, it is easy to see that (9) is the actual condition (26). For (10), it is equivalent to ${\bar{P}}_{i}<\lambda_{i2}I$ and ${\bar{P}}_{i}<\lambda_{i2}I$, that is,

(36) $$\begin{array}{c} -\lambda_{i2}I+R_{i}^{-\frac{1}{2}}P_{i}R_{i}^{-\frac{1}{2}}<0, \end{array}$$

and

(37) $$\begin{array}{c} \lambda_{i1}I-R_{i}^{-\frac{1}{2}}P_{i}R_{i}^{-\frac{1}{2}}<0. \end{array}$$

According to Lemma 2, (26) is equivalent to (36), and (37) is acquired by (27) and (30). From Theorem 1, if $Q_{i}=\gamma^{2}I$, it is concluded that (14) and (35) are equivalent. The rest is similar to the proof of (16)–(21), which is obtained by conditions (9), (10), and (28). This completes the proof. □

**Remark** **4.** *Compared with the literature [42,43,44], controller (22) combines two traditional controllers, u(t)=Kx(t) and $u\left(t\right)=K_{\tau }\left(t\right)x\left(t-\tau \right)$, and therefore is more general and has broader applications, such as networked control systems [45].*

With the idea behind controller (22), another stabilizing controller without a Bernoulli variable is devised

(38) $$u\left(t\right)=K_{\tau }\left(\sigma_{t}\right)x\left(t-\tau \right)+K\left(\sigma_{t}\right)x\left(t\right).$$

Using controller (38) in system (1), which includes the Bernoulli variable, one obtains

$$\left\{\begin{array}{c} \begin{array}{cc} dx(t)= & [S\left(\sigma_{t}\right)x\left(t\right)+S_{\tau }\left(\sigma_{t}\right)x\left(t-\tau \right)+G\left(\sigma_{t}\right)v\left(t\right)+\delta \left(t\right)L\left(\sigma_{t}\right)u\left(t\right)]dt\\ \; & +[U\left(\sigma_{t}\right)x\left(t\right)+U_{\tau }\left(\sigma_{t}\right)x\left(t-\tau \right)+\left(1-\delta \left(t\right))J\left(\sigma_{t}\right)u\left(t\right)+F\left(\sigma_{t}\right)v\left(t\right)\right]d\omega \left(t\right),\\ z(t)= & H\left(\sigma_{t}\right)x\left(t\right)+H_{\tau }\left(\sigma_{t}\right)x\left(t-\tau \right)+D\left(\sigma_{t}\right)v\left(t\right),\;\forall t\in \left[0,\tilde{T}\right],\\ x(t)= & \psi \left(t\right),\;\sigma_{t}=t_{0},\;\forall t\in \left[-\tau ,0\right],\; \end{array} \end{array}\right.$$

which is rewritten as follows

(39) $$\begin{array}{c} \left\{\begin{array}{c} \begin{array}{cc} dx(t)= & [\hat{S}\left(\sigma_{t}\right)x\left(t\right)+{\bar{S}}_{\tau }\left(\sigma_{t}\right)x\left(t-\tau \right)+G\left(\sigma_{t}\right)v\left(t\right)+\left(\delta \left(t\right)-\delta \right)\bar{W}\left(\sigma_{t}\right)]dt\\ & +\left[\bar{U}\left(\sigma_{t}\right)x\left(t\right)+{\hat{U}}_{\tau }\left(\sigma_{t}\right)x\left(t-\tau \right)+F\left(\sigma_{t}\right)v\left(t\right)+\left(\delta \left(t\right)-\delta \right)\bar{Z}\left(\sigma_{t}\right)\right]d\omega \left(t\right),\\ z(t)= & H\left(\sigma_{t}\right)x\left(t\right)+H_{\tau }\left(\sigma_{t}\right)x\left(t-\tau \right)+D\left(\sigma_{t}\right)v\left(t\right),\;\forall t\in \left[0,\tilde{T}\right],\\ x(t)= & \psi \left(t\right),\;\sigma_{t}=t_{0},\;\forall t\in \left[-\tau ,0\right], \end{array} \end{array}\right. \end{array}$$

where

$$\begin{array}{cc} {\bar{S}}_{\tau }\left(\sigma_{t}\right)= & \;S_{\tau }\left(\sigma_{t}\right)+\delta L\left(\sigma_{t}\right)K_{\tau }\left(\sigma_{t}\right),\\ \bar{W}\left(\sigma_{t}\right)= & \;L\left(\sigma_{t}\right)K\left(\sigma_{t}\right)x\left(t\right)+L\left(\sigma_{t}\right)K_{\tau }\left(\sigma_{t}\right)x\left(t-\tau \right),\\ \bar{U}\left(\sigma_{t}\right)= & \;U\left(\sigma_{t}\right)+\left(1-\alpha \right)J\left(\sigma_{t}\right)K\left(\sigma_{t}\right),\\ \bar{Z}\left(\sigma_{t}\right)= & \;J\left(\sigma_{t}\right)K\left(\sigma_{t}\right)x\left(t\right)+J\left(\sigma_{t}\right)K_{\tau }\left(\sigma_{t}\right)x\left(t-\tau \right). \end{array}$$

The following theorem is developed, which is a sufficient condition of $H_{\infty }$ FTB for the closed-loop system (39).

**Theorem** **3.** *System (39) is $H_{\infty }$ FTB with respect to $\left(c_{1},c_{2},\tilde{T},R_{i},d,\gamma \right)$, if for a real scalar η≥0, there exist the constants γ>0, $\lambda_{i1}>0$, $\lambda_{i2}>0$, the symmetric matrix $X_{i}>0$, the matrices ${\bar{O}}_{i}>0$ and $Y_{i}$, $Y_{\tau i}$ satisfying (25)–(28), and*

(40) $$\left[\begin{array}{ccccccc} {\tilde{\Psi }}_{i1} & {\hat{\Psi }}_{i2} & G_{i}X_{i} & H_{i}^{T} & {\hat{\Psi }}_{i3} & {\tilde{\Psi }}_{i4} & X_{i}\\ {}* & {\tilde{\Psi }}_{i5} & 0 & H_{\tau i}^{T} & {\tilde{\Psi }}_{i6} & {\hat{\Psi }}_{i7} & 0\\ {}* & * & -\gamma^{2}I & D_{i} & F_{i}^{T} & 0 & 0\\ {}* & * & * & -I & 0 & 0 & 0\\ {}* & * & * & * & -X_{i} & 0 & 0\\ {}* & * & * & * & * & -X_{i} & 0\\ {}* & * & * & * & * & * & -{\bar{O}}_{i} \end{array}\right]<0,$$

*where*

$$\begin{array}{cc} {\hat{\Psi }}_{i2}= & \;S_{\tau i}X_{i}+\delta L_{i}Y_{\tau i},\;{\hat{\Psi }}_{i7}=\beta Y_{\tau i}^{T}J_{i}^{T},\;{\hat{\Psi }}_{i3}=X_{i}U_{i}^{T}+\left(1-\delta \right)Y_{i}^{T}J_{i}^{T}. \end{array}$$

*The gains of controller (38) are presented by*

$$K_{i}=Y_{i}X_{i}^{-1},\;K_{\tau i}=Y_{\tau i}X_{i}^{-1}.$$

**Proof.**  Choosing the Lyapunov functional (12) for system (39), then $LV(x_{t},\sigma_{t}=i)$ satisfies

(41) $$\begin{array}{c} \begin{array}{cc} & LV(x_{t},\sigma_{t}=i)\\ = & \;x^{T}\left(t\right)\left[{\left(P_{i}{\hat{S}}_{i}\right)}^{🟉}+\sum_{j=1}^{N}\pi_{ij}P_{j}\right]x\left(t\right)+2x^{T}\left(t\right)P_{i}{\bar{S}}_{\tau i}x\left(t-\tau \right)\\ & +2x^{T}\left(t\right)P_{i}G_{i}v\left(t\right)+\beta^{2}{\bar{Z}}_{i}^{T}P_{i}{\bar{Z}}_{i}+x^{T}\left(t\right)O_{i}x\left(t\right)\\ & +{\hat{\Xi }}^{T}P_{i}\hat{\Xi }-x^{T}\left(t-\tau \right)O_{i}x\left(t-\tau \right)\\ = & \;x^{T}\left(t\right)\left[\left(1+\pi_{k}^{i}\right){\left(P_{i}{\hat{S}}_{i}\right)}^{🟉}+\zeta_{k}^{i}+\sum_{j\in L_{uk}^{i}}\pi_{ij}\left({\left(P_{i}{\hat{S}}_{i}\right)}^{🟉}+P_{j}\right)+O_{i}\right]x\left(t\right)\\ & +2x^{T}\left(t\right)P_{i}{\bar{S}}_{\tau i}x\left(t-\tau \right)+\beta^{2}{\bar{Z}}_{i}^{T}P_{i}{\bar{Z}}_{i}+{\hat{\Xi }}^{T}P_{i}\hat{\Xi }\\ & +2x^{T}\left(t\right)P_{i}G_{i}v\left(t\right)-x^{T}\left(t-\tau \right)O_{i}x\left(t-\tau \right), \end{array} \end{array}$$

where $\hat{\Xi }=F_{i}v\left(t\right)+{\tilde{U}}_{i}x\left(t\right)+{\hat{U}}_{\tau i}x\left(t-\tau \right)$.

The next steps are the same as those for the proof of Theorem 2. Pre- and post-multiply (40) by diag $\left\{X_{i}^{-1},X_{i}^{-1},I,I,\cdots ,I\right\}$ and its transpose, respectively. Then, by Schur’s complement and pre- and post-multiplying both sides by $\left[x^{T}\left(t\right)\;x^{T}\left(t-\tau \right)\;v^{T}\left(t\right)\;z^{T}\left(t\right)\right]$ and its transpose, respectively, and, by comparing it with (41), one obtains

$$\begin{array}{c} LV\left(x_{t},\sigma_{t}=i\right)<\eta V\left(x_{t},\sigma_{t}=i\right)+\gamma^{2}v^{T}\left(t\right)v\left(t\right)-z^{T}\left(t\right)z\left(t\right). \end{array}$$

The following step is similar to Theorem 2 and is omitted here. The proof ends. □

**Remark** **5.** *If $K_{\tau }\left(\sigma_{t}\right)=0$, then Theorem 3 is reduced to Theorem 3.3 in [51].*

For system (1), another controller experiencing a disordering phenomenon is described as

(42) $$\begin{array}{c} \begin{array}{cc} u(t)= & \left[\left(1-\delta \left(t\right)\right)K_{\tau }\left(\sigma_{t}\right)+\delta \left(t\right)K\left(\sigma_{t}\right)\right]x\left(t\right)\\ & +\left[\left(1-\delta \left(t\right)\right)K\left(\sigma_{t}\right)+\delta \left(t\right)K_{\tau }\left(\sigma_{t}\right)\right]x\left(t-\tau \right), \end{array} \end{array}$$

which implies

$$\begin{array}{c} \begin{array}{c} u\left(t\right)=\left\{\begin{array}{c} K_{\tau }\left(\sigma_{t}\right)x\left(t-\tau \right)+K\left(\sigma_{t}\right)x\left(t\right),\\ \;\mathrm{if}\;\delta (t)=1\;\mathrm{or}\;\mathrm{without}\;\mathrm{disordering},\\ K\left(\sigma_{t}\right)x\left(t-\tau \right)+K_{\tau }\left(\sigma_{t}\right)x\left(t\right),\\ \;\mathrm{if}\;\delta (t)=0\;\mathrm{or}\;\mathrm{with}\;\mathrm{disordering}. \end{array}\right. \end{array} \end{array}$$

It is easy to see that (42) is the same as

(43) $$\begin{array}{c} \begin{array}{cc} u(t)= & \left[\left(1-\delta \right)K_{\tau }\left(\sigma_{t}\right)+\delta K\left(\sigma_{t}\right)+\left(\delta \left(t\right)-\delta \right)\left(K\left(\sigma_{t}\right)-K_{\tau }\left(\sigma_{t}\right)\right)\right]x\left(t\right)\\ & +\left[\left(1-\delta \right)K\left(\sigma_{t}\right)+\delta K_{\tau }\left(\sigma_{t}\right)+\left(\delta \left(t\right)-\delta \right)\left(K_{\tau }\left(\sigma_{t}\right)-K\left(\sigma_{t}\right)\right)\right]x\left(t-\tau \right). \end{array} \end{array}$$

Controller (43) is applied to system (1), and let $\delta_{t}=\delta \left(t\right)-\delta$. Then, we have

(44) $$\begin{array}{c} \left\{\begin{array}{cc} dx(t)= & \;[\tilde{S}\left(\sigma_{t}\right)x\left(t\right)++{\tilde{S}}_{\tau }\left(\sigma_{t}\right)x\left(t-\tau \right)+Gv\left(t\right)\\ & +\delta_{t}L\left(\sigma_{t}\right)\left(K\left(\sigma_{t}\right)-K_{\tau }\left(\sigma_{t}\right)\right)x\left(t\right)\\ & +\delta_{t}L\left(\sigma_{t}\right)\left(K_{\tau }\left(\sigma_{t}\right)-K\left(\sigma_{t}\right)\right)x\left(t-\tau \right)]dt\\ & +[\tilde{U}\left(\sigma_{t}\right)x\left(t\right)+Fv\left(t\right)+{\tilde{U}}_{\tau }\left(\sigma_{t}\right)x\left(t-\tau \right)\\ & +\delta_{t}J\left(\sigma_{t}\right)\left(K\left(\sigma_{t}\right)-K_{\tau }\left(\sigma_{t}\right)\right)x\left(t\right)\\ & +\delta_{t}J\left(\sigma_{t}\right)\left(K_{\tau }\left(\sigma_{t}\right)-K\left(\sigma_{t}\right)\right)x\left(t-\tau \right)]d\omega ,\\ z(t)= & \;H\left(\sigma_{t}\right)x\left(t\right)+H_{\tau }\left(\sigma_{t}\right)x\left(t-\tau \right)+D\left(\sigma_{t}\right)v\left(t\right),\;\forall t\in \left[0,\tilde{T}\right],\\ x(t)= & \;\psi \left(t\right),\;\sigma_{t}=t_{0},\;\forall t\in \left[-\tau ,0\right], \end{array}\right. \end{array}$$

where

$$\begin{array}{cc} \tilde{S}\left(\sigma_{t}\right)= & \;S\left(\sigma_{t}\right)+L\left(\sigma_{t}\right)\left[\delta K\left(\sigma_{t}\right)+\left(1-\delta \right)K_{\tau }\left(\sigma_{t}\right)\right],\\ {\tilde{S}}_{\tau }\left(\sigma_{t}\right)= & \;S_{\tau }\left(\sigma_{t}\right)+L\left(\sigma_{t}\right)\left[\delta K_{\tau }\left(\sigma_{t}\right)+\left(1-\delta \right)K\left(\sigma_{t}\right)\right],\\ \tilde{U}\left(\sigma_{t}\right)= & \;U\left(\sigma_{t}\right)+J\left(\sigma_{t}\right)\left[\delta K\left(\sigma_{t}\right)+\left(1-\delta \right)K_{\tau }\left(\sigma_{t}\right)\right],\\ {\tilde{U}}_{\tau }\left(\sigma_{t}\right)= & \;U_{\tau }\left(\sigma_{t}\right)+J\left(\sigma_{t}\right)\left[\delta K_{\tau }\left(\sigma_{t}\right)+\left(1-\delta \right)K\left(\sigma_{t}\right)\right]. \end{array}$$

Then, the following theorem is developed.

**Theorem** **4.** *For the given real scalar η≥0, system (44) is $H_{\infty }$ FTB with respect to $\left(c_{1},c_{2},\tilde{T},R_{i},d,\gamma \right)$, if there exist γ>0, $\lambda_{i1}>0$, $\lambda_{i2}>0$, the matrices $X_{i}>0$, ${\bar{O}}_{i}>0$ and $Y_{i}$, $Y_{\tau i}$ satisfying (25)–(28), and*

(45) $$\left[\begin{array}{ccccccc} {\overset{˘}{\Psi }}_{i1} & {\overset{˘}{\Psi }}_{i2} & G_{i}X_{i} & H_{i}^{T} & {\overset{˘}{\Psi }}_{i3} & {\overset{˘}{\Psi }}_{i4} & X_{i}\\ {}* & {\tilde{\Psi }}_{i5} & 0 & H_{\tau i}^{T} & {\overset{˘}{\Psi }}_{i6} & {\overset{˘}{\Psi }}_{i7} & 0\\ {}* & * & -\gamma^{2}I & D_{i} & F_{i}^{T} & 0 & 0\\ {}* & * & * & -I & 0 & 0 & 0\\ {}* & * & * & * & -X_{i} & 0 & 0\\ {}* & * & * & * & * & -X_{i} & 0\\ {}* & * & * & * & * & * & -{\bar{O}}_{i} \end{array}\right]<0.$$

*where*

$$\begin{array}{cc} {\overset{˘}{\Psi }}_{i1}= & \;\left(1+X_{i}\pi_{k}^{i}\right){\left(S_{i}X_{i}+\delta L_{i}Y_{i}+\left(1-\delta \right)L_{i}Y_{\tau i}\right)}^{🟉}+X_{i}\zeta_{k}^{i}X_{i}^{T}-\eta X_{i}\\ \; & +X_{i}\sum_{j\in L_{uk}^{i}}\pi_{ij}\left[{\left(S_{i}X_{i}+\delta L_{i}Y_{i}+\left(1-\delta \right)L_{i}Y_{\tau i}\right)}^{🟉}+X_{j}^{-1}X_{i}^{T}\right],\\ {\overset{˘}{\Psi }}_{i2}= & \;S_{\tau i}X_{i}+\delta L_{i}Y_{\tau i}+\left(1-\delta \right)L_{i}Y_{i},\;{\overset{˘}{\Psi }}_{i3}=X_{i}U_{i}^{T}+\delta Y_{i}^{T}J_{i}^{T}+\left(1-\delta \right)Y_{\tau i}^{T}J_{i}^{T},\\ {\overset{˘}{\Psi }}_{i4}= & \;\beta \left(Y_{i}^{T}J_{i}^{T}-Y_{\tau i}^{T}J_{i}^{T}\right),\;{\overset{˘}{\Psi }}_{i7}=\beta \left(Y_{\tau i}^{T}J_{i}^{T}-Y_{i}^{T}J_{i}^{T}\right),\\ {\overset{˘}{\Psi }}_{i6}= & \;X_{i}U_{\tau i}^{T}+\delta Y_{\tau i}^{T}J_{i}^{T}+\left(1-\delta \right)Y_{i}^{T}J_{i}^{T}. \end{array}$$

*Then, the gains of controller (42) are obtained by*

$$K_{i}=Y_{i}X_{i}^{-1},\;K_{\tau i}=Y_{\tau i}X_{i}^{-1}.$$

**Proof.**  Choosing the Lyapunov functional (12) for system (44), it is obtained that

(46) $$\begin{array}{c} \begin{array}{cc} & LV(x_{t},\sigma_{t}=i)\\ = & \;x^{T}\left(t\right)\left[{\left(P_{i}{\tilde{S}}_{i}\right)}^{🟉}+\sum_{j=1}^{N}\pi_{ij}P_{j}\right]x\left(t\right)+\beta^{2}{\overset{˘}{Z}}_{i}^{T}P_{i}{\overset{˘}{Z}}_{i}\\ & +2x^{T}\left(t\right)P_{i}G_{i}v\left(t\right)+2x^{T}\left(t\right)P_{i}{\tilde{S}}_{\tau i}x\left(t-\tau \right)\\ & +x^{T}\left(t\right)O_{i}x\left(t\right)+{\overset{˘}{\Xi }}^{T}P_{i}\overset{˘}{\Xi }-x^{T}\left(t-\tau \right)O_{i}x\left(t-\tau \right)\\ = & \;x^{T}\left(t\right)[\left(1+\pi_{k}^{i}\right){\left(P_{i}{\tilde{S}}_{i}\right)}^{🟉}+\zeta_{k}^{i}+P_{j}+O_{i}]x\left(t\right)\\ & +2x^{T}\left(t\right)P_{i}{\tilde{S}}_{\tau i}x\left(t-\tau \right)+2x^{T}\left(t\right)P_{i}G_{i}v\left(t\right)\\ & +\beta^{2}{\overset{˘}{Z}}_{i}^{T}P_{i}{\overset{˘}{Z}}_{i}+{\overset{˘}{\Xi }}^{T}P_{i}\overset{˘}{\Xi }-x^{T}\left(t-\tau \right)O_{i}x\left(t-\tau \right). \end{array} \end{array}$$

where

$$\begin{array}{cc} \overset{˘}{\Xi }= & \;{\tilde{U}}_{i}x\left(t\right)+{\tilde{U}}_{\tau i}x\left(t-\tau \right)+F_{i}v\left(t\right),\\ {\overset{˘}{Z}}_{i}= & \;J_{i}\left(K_{i}-K_{\tau i}\right)x\left(t\right)+J_{i}\left(K_{\tau }i-K_{i}\right)x\left(t-\tau \right). \end{array}$$

Pre- and post-multiply (45), respectively, by diag $\left\{X_{i}^{-1},X_{i}^{-1},I,I,\cdots ,I\right\}$ and its transpose. Then, from Lemma 2, by pre- and post-multiplying both sides by $\left[x^{T}\left(t\right)\;x^{T}\left(t-\tau \right)\;v^{T}\left(t\right)\;z^{T}\left(t\right)\right]$ and its transpose, respectively, and comparing it with (46), one obtains

$$\begin{array}{c} LV\left(x_{t},\sigma_{t}=i\right)<\eta V\left(x_{t},\sigma_{t}=i\right)+\gamma^{2}v^{T}\left(t\right)v\left(t\right)-z^{T}\left(t\right)z\left(t\right). \end{array}$$

The next steps are same as Theorem 2 and are omitted here. The proof is complete. □

**Remark** **6.** *If δ(t)=1 or controller (42) does not experience a disordering phenomenon, then Theorem 4 is reduced to Theorem 3.*

## 4. Numerical Examples

In this part, three examples are given to illustrate the effectiveness of the proposed results.

**Example** **1.** *Consider system (1) with the following parameters:*

Mode1:

$$\begin{array}{c} S_{1}=\left[\begin{array}{cc} -3.1 & 0.3\\ 1 & 0.1 \end{array}\right],\;S_{\tau 1}=\left[\begin{array}{cc} -1.7 & 1.1\\ 0 & 0.2 \end{array}\right],\;U_{1}=\left[\begin{array}{cc} 0.61 & 0.13\\ 0.17 & 0.15 \end{array}\right],\;U_{\tau 1}=\left[\begin{array}{cc} -0.2 & 0.1\\ 0 & -0.1 \end{array}\right],\; \end{array}$$

$$\begin{array}{c} G_{1}=\left[\begin{array}{cc} 0.1 & -1.3\\ 0.2 & -0.9 \end{array}\right],\;F_{1}=\left[\begin{array}{cc} -0.2 & -0.7\\ 1.9 & 0 \end{array}\right],\;L_{1}=\left[\begin{array}{c} 2.1\\ 0.9 \end{array}\right],\;J_{1}=\left[\begin{array}{c} 1.1\\ 0.5 \end{array}\right],\;R_{1}=\left[\begin{array}{cc} 1 & 0\\ 0 & 1 \end{array}\right],\; \end{array}$$

$$\begin{array}{c} \;H_{1}=\left[\begin{array}{cc} -0.4 & 0.1\\ 0.7 & 0.1 \end{array}\right],\;H_{\tau 1}=\left[\begin{array}{cc} 0.1 & 0.2\\ 0.2 & -0.3 \end{array}\right],\;D_{1}=\left[\begin{array}{cc} -0.2 & 0.5\\ 0.3 & -0.1 \end{array}\right]. \end{array}$$

Mode2:

$$\begin{array}{c} S_{2}=\left[\begin{array}{cc} -3.9 & 0.9\\ 1.1 & 0 \end{array}\right],\;S_{\tau 2}=\left[\begin{array}{cc} -0.7 & 1.2\\ 0 & 0.3 \end{array}\right],\;U_{2}=\left[\begin{array}{cc} 0.5 & 0.3\\ 0.1 & 0.3 \end{array}\right],\;U_{\tau 2}=\left[\begin{array}{cc} -0.2 & 0.3\\ 0.5 & -0.1 \end{array}\right],\; \end{array}$$

$$\begin{array}{c} G_{2}=\left[\begin{array}{cc} 0.3 & -0.9\\ 0.7 & -1.1 \end{array}\right],\;F_{2}=\left[\begin{array}{cc} -0.1 & -1\\ 0.2 & 0.1 \end{array}\right],\;L_{2}=\left[\begin{array}{c} 1.6\\ 1.5 \end{array}\right],\;J_{2}=\left[\begin{array}{c} 1.3\\ 0.4 \end{array}\right],\;R_{2}=\left[\begin{array}{cc} 1 & 0\\ 0 & 1 \end{array}\right],\; \end{array}$$

$$\begin{array}{c} \;H_{2}=\left[\begin{array}{cc} -0.3 & 0.1\\ 0.9 & 0.3 \end{array}\right],\;H_{\tau 2}=\left[\begin{array}{cc} 0.1 & 0.2\\ 0.1 & -0.3 \end{array}\right],\;D_{2}=\left[\begin{array}{cc} -0.2 & 0.6\\ 0.2 & -0.1 \end{array}\right]. \end{array}$$

The partially unknown transition rate matrix is

$$\begin{array}{c} \Pi =\left[\begin{array}{cc} -0.5 & 0.5\\ ? & ? \end{array}\right].\; \end{array}$$

**Figure 1:**
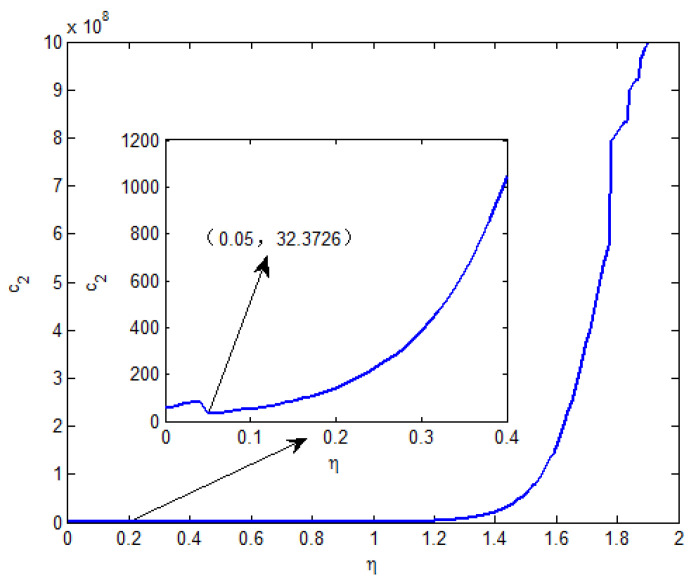
When η∈[0,1.90], the curve of $c_{2}$.

**Figure 2:**
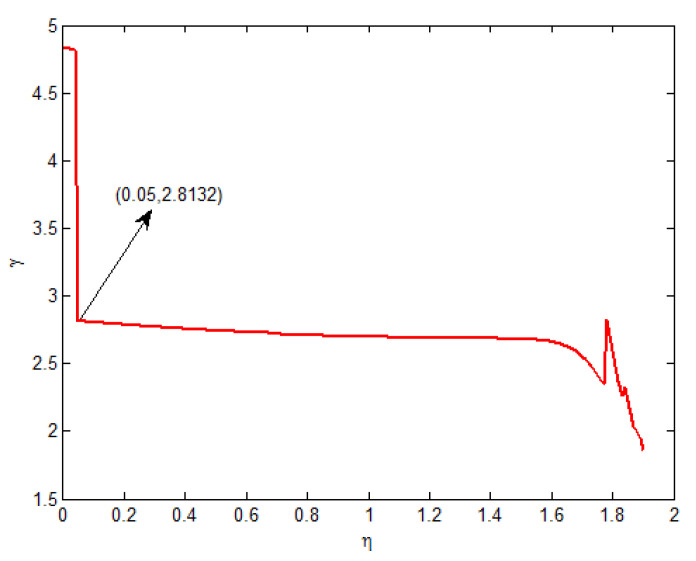
When η∈[0,1.90], the curve of γ.

Moreover, $\tilde{T}=10,c_{1}=0.5,\tau =1$, $\delta =0.6,d=1,x_{0}={\left[0.1\;-0.05\right]}^{T}$, and $v\left(t\right)=\frac{1}{\left(1\;+\;t^{2}\right)}$. From Theorem 2, the feasible solution can be found when η∈[0,1.90]. The relationship curves of η with $c_{2}$ and γ are shown in Figure 1 and Figure 2, respectively. From Figure 1, it is seen that the minimum value of $c_{2}$ is 32.3726 and the corresponding γ=2.8132 when η=0.05.

When η=0.05, the gains of controller (22) are

$$\begin{array}{c} K_{1}=\left[\begin{array}{cc} -1.8478 & -1.1423 \end{array}\right],K_{\tau 1}=\left[\begin{array}{cc} 0.1438 & -0.2441 \end{array}\right],\; \end{array}$$

$$\begin{array}{c} K_{2}=\left[\begin{array}{cc} -1.1312 & -1.4924 \end{array}\right],K_{\tau 2}=\left[\begin{array}{cc} 0.0100 & -0.2335 \end{array}\right].\; \end{array}$$

This indicates that under controller (22), when $E[x^{T}\left(t_{1}\right)R_{i}x\left(t_{1}\right)]\leq 0.5$, $t_{1}\in \left[-1,0\right]$, then $E[x^{T}\left(t_{2}\right)R_{i}x\left(t_{2}\right)]<32.3726$, $t_{2}\in \left[0,1\right]$, and $E\left[\int_{0}^{1}z^{T}\left(t\right)z\left(t\right)dt\right]<2.8132^{2}E\left[\int_{0}^{1}v^{T}\left(t\right)v\left(t\right)dt\right]$.

**Figure 3:**
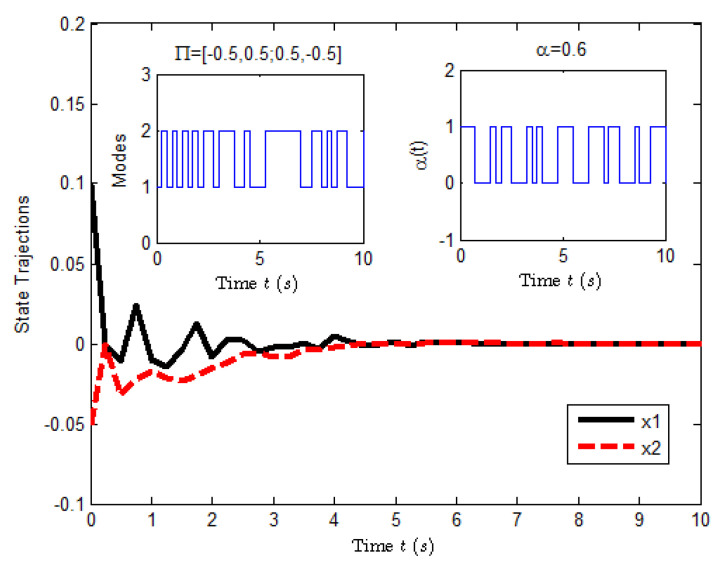
The state response of system (23).

**Figure 4:**
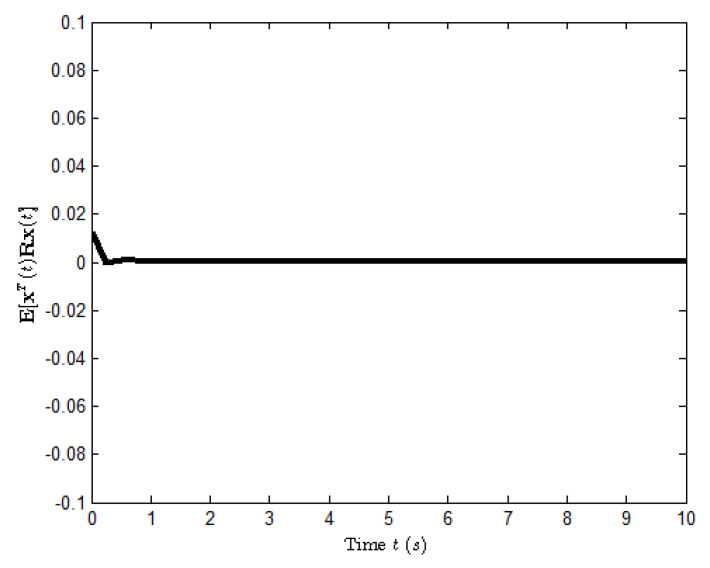
The evolution of $E[x^{T}\left(t\right)Rx\left(t\right)]$ for system (23).

According to the conditions mentioned above, Figure 3 shows the state response of system (23), where the small figures represent the curves of a possible Markovian mode evolution and the evolution of the Bernoulli variable δ(t) with δ=0.6. The evolution of $E[x^{T}\left(t\right)Rx\left(t\right)]$ is shown in Figure 4, which implies that the closed-loop system (23) is $H_{\infty }$ FTB.

**Figure 5:**
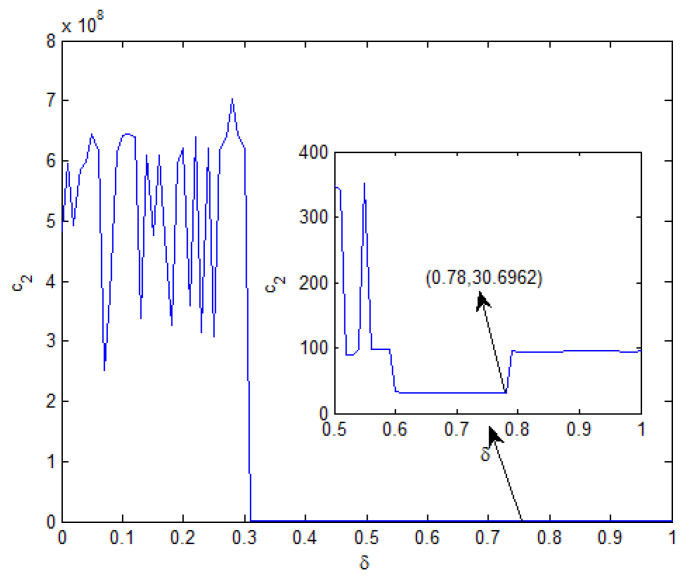
The relationship between δ and $c_{2}$.

In order to show the advantages of Theorem 2 and the influence of the probability δ, Figure 5 depicts the relationship between $c_{2}$ and δ. It is seen that $c_{2}$ takes the minimum value when δ=0.78. This means controller (22) has less conservatism.

**Example** **2.** *Consider system (39) with the parameters of Example 1. By Theorem 3, we obtain the feasible solution when η∈[0,1.90]. The minimum value of $c_{2}$ is 67.8162 and the corresponding γ=5.0148 when η=0. The gains of controller (38) are*

$$\begin{array}{c} K_{1}=\left[\begin{array}{cc} 2.1007 & -1.3952 \end{array}\right],K_{\tau 1}=\left[\begin{array}{cc} 0.3184 & -0.1772 \end{array}\right],\; \end{array}$$

$$\begin{array}{c} K_{2}=\left[\begin{array}{cc} -0.2674 & -1.8616 \end{array}\right],K_{\tau 2}=\left[\begin{array}{cc} 0.1328 & -0.4017 \end{array}\right].\; \end{array}$$

**Figure 6:**
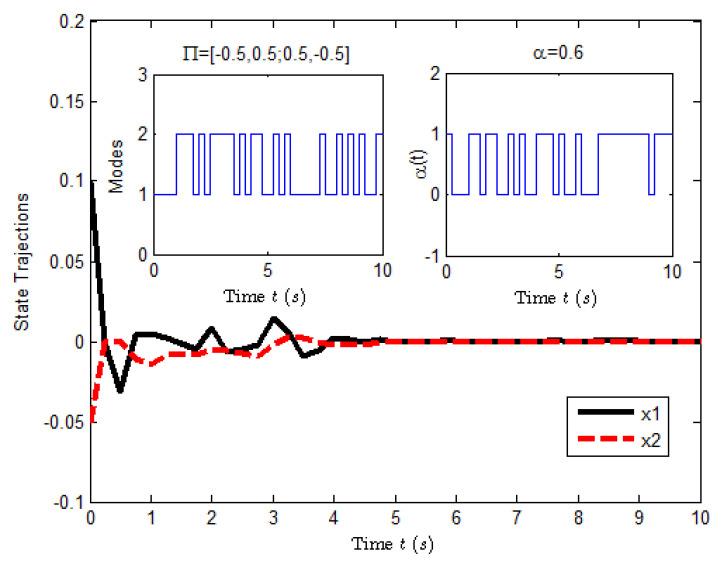
The state response of system (39).

**Figure 7:**
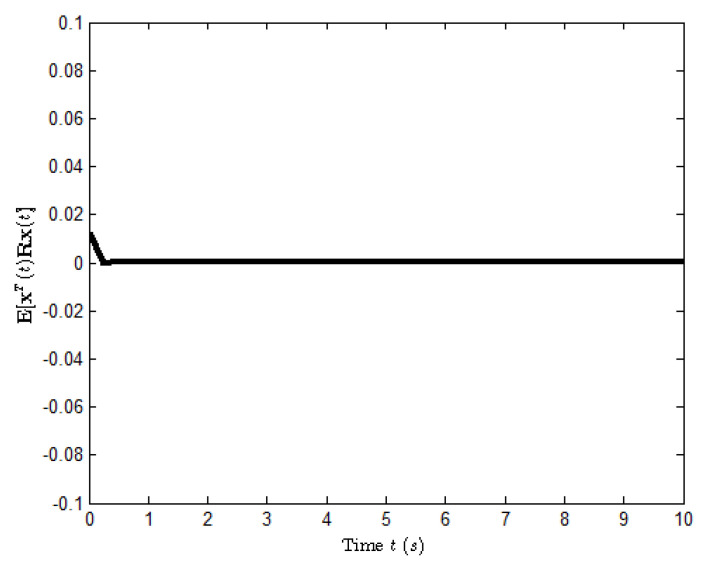
The evolution of $E[x^{T}\left(t\right)Rx\left(t\right)]$ for system (39).

Figure 6 and Figure 7 show the state response of system (39) and the evolution of $E[x^{T}\left(t\right)Rx\left(t\right)]$, respectively. From these figures, it is seen that the closed-loop system (39) is $H_{\infty }$ FTB by the designed controller (38). This implies that Theorem 3 is valid.

**Example** **3.** *Consider system (44) with the system parameters of Example 1. By Theorem 4, the feasible region is η∈[0,1.89]. When η=0.03, the minimum value of $c_{2}$ is 310.9813, and the corresponding γ=9.7316. The gains of controller (42) are*

$$\begin{array}{c} K_{1}=\left[\begin{array}{cc} -0.5970 & -0.3385 \end{array}\right],K_{\tau 1}=\left[\begin{array}{cc} 0.8791 & -0.2409 \end{array}\right],\; \end{array}$$

$$\begin{array}{c} K_{2}=\left[\begin{array}{cc} -0.6966 & -0.3475 \end{array}\right],K_{\tau 2}=\left[\begin{array}{cc} 0.6689 & -0.2812 \end{array}\right].\; \end{array}$$

**Figure 8:**
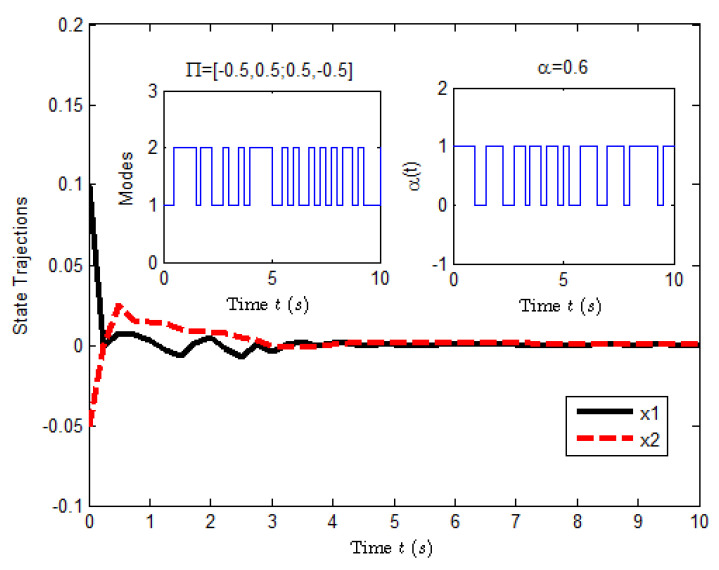
The state response of system (44).

**Figure 9:**
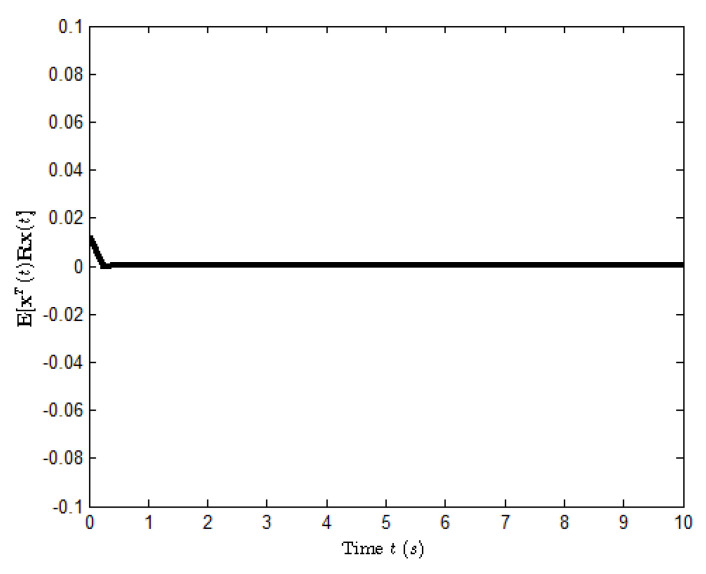
The evolution of $E[x^{T}\left(t\right)Rx\left(t\right)]$ for system (44).

Similar to Example 2, the state response of system (44) is shown in Figure 8, and the evolution of $E[x^{T}\left(t\right)Rx\left(t\right)]$ is drawn in Figure 9. It is concluded from these plots that the closed-loop system (44) is $H_{\infty }$ FTB, by the designed controller (42). Therefore, Theorem 4 is valid.

## 5. Conclusions

In this paper, the FTB and $H_{\infty }$ FTB problems of time-delay Markovian jump systems with a partially unknown transition rate have been studied. A sufficient condition of FTB for the given system is obtained by the LMIs technique and the Lyapunov functional method. A new controller that is partially time delay-dependent is designed. This controller has the advantages of strong generality and less conservative property. Based on PDDCs, two new kinds of controllers are derived; one does not contain the Bernoulli variable, and the other describes controllers experiencing a disordering phenomenon. Combined with LMIs, some sufficient conditions of $H_{\infty }$ FTB for closed-loop systems are given via the designed controllers. Three numerical examples illustrate that the proposed methods are effective. The results in this paper can be extended to the $H_{\infty }$ filtering problem for Markovian jump systems with time-varying delays. In the future, the FTB and $H_{\infty }$ FTB problems of fractional systems will be considered by means of the theories of fractional calculus and negative probabilities [56].

## Data Availability

Not applicable.

## Appendix A

In this section, Lemma 1 is reformulated with sharp inequalities and also proved.

Let g(t) be a nonnegative continuous function. If there are positive constants r,q such that

(A1) $$\begin{array}{c} \begin{array}{c} g\left(t\right)<r+q\int_{0}^{t}g\left(s\right)ds,\;0\leq t\leq \tilde{T}, \end{array} \end{array}$$

then

(A2) $$\begin{array}{c} \begin{array}{c} g\left(t\right)<r\exp\left(qt\right),\;0\leq t\leq \tilde{T}. \end{array} \end{array}$$

**Proof.** Let

(A3) $$\begin{array}{c} U\left(t\right)=r+q\int_{0}^{t}g\left(s\right)ds,\;0\leq t\leq \tilde{T}. \end{array}$$

Then, the derivative of U(t) is $\dot{U}\left(t\right)=qg\left(t\right)$. From (A1), we have $\dot{U}\left(t\right)<qU\left(t\right)$, i.e., $\dot{U}\left(t\right)-qU\left(t\right)<0$. Then, it is deduced that

$$\dot{U}\left(t\right)\exp\left(-qt\right)-qU\left(t\right)\exp\left(-qt\right)<0,$$

which implies that the derivative of U(t)exp(qt) satisfies ${\left(U\left(t\right)\exp\left(-qt\right)\right)}^{\prime }<0$. From the monotonicity, U(t)exp(−qt)<U(0)=r, which guarantees that U(t)<rexp(qt). Together with (A1) and (A3), (A2) is obtained. □
